# Mandating influenza vaccinations for health care workers: analysing opportunities for policy change using Kingdon’s agenda setting framework

**DOI:** 10.1186/s12913-016-1772-0

**Published:** 2016-09-29

**Authors:** Angela Jackson-Lee, Neil G. Barr, Glen E. Randall

**Affiliations:** McMaster University, Hamilton, ON Canada

**Keywords:** Influenza, Vaccination, Health policy, Health care workers, Agenda setting

## Abstract

**Background:**

The consequences of annual influenza outbreaks are often underestimated by the general public. Influenza poses a serious public health threat around the world, particularly for the most vulnerable populations. Fortunately, vaccination can mitigate the negative effects of this common infectious disease. Although inoculating frontline health care workers (HCWs) helps minimize disease transmission, some HCWs continue to resist participating in voluntary immunization programs. A potential solution to this problem is government-mandated vaccination for HCWs; however, in practice, there are substantial barriers to the adoption of such policies. The purpose of this paper is to identify the likelihood of adopting a policy for mandatory immunization of HCWs in Ontario based on a historical review of barriers to the agenda setting process.

**Methods:**

Documents from secondary data sources were analysed using Kingdon’s agenda setting framework of three converging streams leading to *windows of opportunity* for possible policy adoption.

**Results:**

The problems, politics, and policies streams of Kingdon’s framework have converged and diverged repeatedly over an extended period (policy windows have opened and closed several times). In each instance, a technically feasible solution was available. However, despite the evidence supporting the value of HCW immunization, alignment of the three agenda setting streams occurred for very short periods of time, during which, opposition lobby groups reacted, making the proposed solution less politically acceptable.

**Conclusions:**

Prior to the adoption of any new policies, issues must reach a government’s decision agenda. Based on Kingdon’s agenda setting framework, this only occurs when there is alignment of the problems, politics, and policies streams. Understanding this process makes it easier to predict the likelihood of a policy being adopted, and ultimately implemented. Such learning may be applied to policy issues in other jurisdictions. In the case of mandatory influenza vaccinations for HCWs in Ontario, it seems highly unlikely that a new policy will be adopted until perception of the problem’s importance is sufficient to overcome the political opposition to implementing a solution and thus, create a window of opportunity that is open long enough to support change.

## Background

The consequences of annual influenza outbreaks are often underestimated by the general public. This circumstance may be due in part to a large proportion of healthy young adults experiencing only minor symptoms [1], which has contributed to a lack of public awareness and complacency regarding the severity of its effects on vulnerable populations such as the elderly and individuals that are immunocompromised. Despite this misconception, influenza poses a serious public health threat around the world. The World Health Organization (WHO) estimates that annual influenza epidemics cause 3 to 5 million cases of severe illness worldwide, along with 250,000 to 500,000 annual deaths [2]. These epidemics are also associated with overwhelmed clinics and hospitals, many of which need to account for increased staff absenteeism and productivity losses due to illness of health care workers (HCWs) [2].

The impact of annual influenza epidemics on some of the most susceptible populations, such those living with chronic conditions, is even more striking. Global meta-analyses of risk factors for severe disease from pandemic influenza found that 31 % of patients hospitalized had at least one other chronic medical condition, as did 52 % of those admitted to intensive care units and 62 % of fatal cases [3]. In the United States, individuals who were 65 or older consistently accounted for approximately 90 % of all influenza related deaths between 1976 and 2007 [3].

Fortunately, vaccination can mitigate the negative effects of this common infectious disease. During the influenza seasons of 2010–2012, the Centers for Disease Control and Prevention found that influenza vaccination helped reduce children's risk of influenza-related intensive care unit admissions by 74 % [4]. The use of vaccinations has also been associated with a 77 % reduction in influenza-related hospitalization among adults aged 50 or over [5]. In addition, rates of influenza infections were found to be lower in vaccinated HCWs versus unvaccinated HCWs [6] and other healthy adults [7]. However, while vaccination may reduce the risk of influenza in HCWs, a recent systematic review reported the reduced risk to be less than 50 % as compared to HCWs who are not vaccinated; which suggests the need for additional solutions beyond HCW vaccinations [6]. Furthermore, recent reviews highlight the lack of clear evidence to support the benefit of vaccinating HCWs [8] to prevent the spread of influenza to elderly [1].

Within high-income countries, influenza vaccination is readily accessible and many jurisdictions have implemented publicly funded programs to cover the cost [9]. However, public vaccination uptake has been variable [10, 11], and perhaps more importantly, some HCWs continue to resist participation in vaccination programs. For example, in Canada, voluntary uptake of the seasonal influenza vaccine by HCWs remains below the 90 % recommended level [12] and varies substantially across health care organizations [13]. Other studies have reported that in some locations more than 50 % of physicians also fail to take advantage of the annual influenza vaccination [13]. Inadequate levels of HCW immunization can place the vulnerable populations they care for at greater risk of health complications [14].

A potential solution to this problem is government-mandated inoculation for HCWs. However, in practice, there are substantial barriers to the adoption of such policies. For instance, the attitudes of HCWs in the United States toward whether to be vaccinated against influenza or not remains divided, prompting a backlash against suggestions of any mandatory policies [15]. Surveys have revealed multiple reasons why HCWs disapprove of mandated vaccination. These include beliefs that the decision to be vaccinated is personal, fears of side effects, and concern that influenza vaccines are ineffective [15]. There have also been legal obstacles to mandating vaccination as worker unions and professional associations have argued that such policies violate individual rights and freedoms [16]. Specifically, in Canada, it has been argued that mandated immunization violates one's right to refuse unwanted medical treatment under section 7 of the Canadian Charter of Rights and Freedoms [16].

New public policies are rarely adopted based solely on supportive evidence, public opinion, or interest group lobbying; rather, they typically require a confluence of events that is difficult to predict or orchestrate. Through the use of relevant frameworks, theories, or models, policy analysis can provide a greater understanding of the various processes involved and, in turn, enhance the likelihood of recognizing and seizing opportunities for new policies to be adopted [17]. In this vein, Kingdon’s [18] three process streams framework helps to explain how three elements or “streams”—*problems*, *politics*, and *policies*—affect policy agenda setting dynamics. Kingdon argues that, while there is some interplay among the streams, they are largely independent of one another [18]. This framework can be used to explain how issues may make their way onto the government’s decision agenda, which is a key step in the policy adoption process. The purpose of this paper is to identify the likelihood of adopting a policy for mandatory immunization of HCWs in Ontario based on a historical review of barriers to the agenda setting process.

## Methods

Background information regarding the effectiveness and uptake of influenza vaccinations was gathered via narrative review, which included academic and newspaper articles, as well as government reports. Subsequently, these materials were analysed using Kingdon’s agenda setting framework of three converging streams leading to *windows of opportunity* for possible policy adoption. No permissions to analyse the data used in this study were necessary or granted.

The authors independently categorized historical events as falling primarily into the problems, politics, or policies stream (see Table 1). Categories are based on Kingdon’s description as noted below. Any discrepancies in classification were resolved through discussion.

**Table 1:** Windows of opportunity for policy change (mandatory vaccination of health care workers)

	1999–2002	2006–2009	2010–2015
Problems (perception there is a problem that needs to be acted addressed)	▪ ER overcrowding due to heavy flu season resulting in reduced access to care	▪ SARS (severe acute respiratory syndrome) outbreak ▪ H1N1 outbreak/pandemic	▪ Growing calls from experts for mandatory HCW immunization
Politics (events promoting or inhibiting political action)	▪ Heightened media attention pressures government to act ▪ Court challenges in opposition to mandatory HCW vaccination	▪ Production delays, low vaccine supplies and rationing ▪ H1N1 outbreak turns out to be mild (loss of public interest)	▪ Evidence of cost-effectiveness of flu vaccination ▪ Other jurisdictions adopting mandatory policies for HCWs
Policies (availability and feasibility of options)	▪ Wide availability of public vaccination programs	▪ Favorable arbitrator ruling on mandatory HCW vaccinations	▪ Favorable court rulings on mandatory HCW vaccinations

In Kingdon’s framework, the problems stream involves the identification of a particular social problem that has gained public or decision-maker attention and cannot be easily ignored. These problems may come to light through awareness of a change in an indicator, such as an increased infection rate, or a focusing event/crisis, such as the Severe Acute Respiratory Syndrome (SARS) outbreak in 2003. In the politics stream, the governmental agenda is formulated and the list of issues or problems to be given attention is prioritized. This stream is impacted by political events (such as changes in public opinion/national mood), and organized forces (such as a change in government due to elections). In the policies stream, experts analyse the various problems, and suggest technically feasible and politically acceptable solutions to them.

Generally, the three streams flow independently of one another; however, a time-limited *window of opportunity* may open when these streams converge (as politically acceptable solutions to prioritized problems are identified). At this point of convergence, issues are most likely to reach the government’s decision agenda—where policy problems and their proposed solutions are under active discussion by government decision-makers—and thus, most likely to result in the adoption of a new policy. Policy *windows* can also close rapidly. This tends to occur when: problems, politics, and policies are not adequately linked together; initiatives that are implemented lead to either failure or success; indicators of severity lessen; other critical items push the issue off the agenda; there has been movement through an “issue attention cycle” (i.e., a gradual decline of interest in the condition).

Using Kingdon’s agenda setting framework (three process streams that lead to windows of opportunity when they converge) the objective of this paper is to analyse the likelihood of government adopting a mandatory vaccination policy for HCWs in Ontario.

## Results and discussion

### 1999–2002

Ontario endured through a difficult 1999-2000 influenza season. Hospital emergency departments experienced serious overcrowding [19], which resulted in less access to health services for patients seeking critical care. This unfavourable set of circumstances was a focusing event/crisis (problems stream) that resulted in heightened media attention and highlighted the need for government action (politics stream) [19]. During this period, an effective influenza vaccine that had been used as part of a public program in other jurisdictions was available, suggesting that mandatory vaccination was a technically viable and politically acceptable solution (policies stream).

In July 2000 it was announced that Ontario would be the first province in the country to offer free influenza vaccinations to all citizens for the upcoming season to relieve the aforementioned pressure on emergency departments [19]. Ontario’s Universal Influenza Immunization Program (UIIP) is offered annually through the Ministry of Health and Long-Term Care (MOHLTC), with the goal of reducing the number of influenza cases and the associated negative impact on the health care system. All individuals who live, work, or attend school in the province, and are 6 months of age or older, are covered by the program [20]. In recent years, UIIP has increased the accessibility of vaccinations by making them available at a variety of locations such as employer-sponsored clinics and pharmacies [20].

By immunizing a critical mass of the general population, as well as HCWs, it was expected that there would be a dramatic reduction to the death toll as well as the social and economic costs associated with annual influenza outbreaks. Studies have shown that since introducing UIIP in Ontario, the number of reported cases of influenza has declined by 61 %, while mortality has been reduced by 28 % [21]. Additionally, reductions in the number of hospitalizations have helped decrease the costs of health care services by 52 % [21]. As a result of changes brought about by UIIP, Ontario exceeded the target coverage rate of 70 % among the elderly in 2005, which surpassed rates from other Canadian provinces and the United States [22]. Despite broad public acceptance and substantial participation in the voluntary immunization program, pockets of HCW resistance persisted (politics stream), and outbreaks in long-term care facilities and hospitals continued to occur, resulting in preventable illness and death [23]. As a consequence, Ontario’s MOHLTC attempted to promote voluntary vaccination of HCWs by requiring health care facilities to report vaccination rates [24].

By winter 2002, UIIP was in its second year and the issue of mandatory vaccination of HCWs was beginning to become controversial and disputed in the courts [25]. When a health care system in Hamilton instituted a mandatory vaccination policy during outbreaks, or work suspension for non-compliance, 15 staff members refused inoculation and were removed from duty without pay. This led to arbitration where the union presented its case as forced medical treatment. The arbitrator agreed with the union position citing that the policy was not supported by regulatory or statutory authority. In the decision, the arbitration board declared a violation of section 7 of the Charter of Rights and Freedoms, which assures security of the person. Mandatory influenza vaccination, the arbitrator decided, was a forced medical act and therefore, contravened the Charter (St. Peter’s Health System v. CUPE local 778, 2002) [25].

Around the same time, a paramedic working in Ontario was making a constitutional challenge against the addition of annual influenza immunization to the Ontario Ambulance Act (North Bay Hospital v. CUPE local 139, 2003) [25]. His challenge also argued that mandatory immunization violated section 7 of the Charter. Pressure from the unions resulted in an amendment to the *Ambulance Act* in 2002, replacing mandatory vaccination with a requirement for influenza education. These cases/political events represented a coordinated force in opposition to the mandatory vaccination initiative (politics stream). As a result, the problems, politics and policies streams were no longer aligned, closing the window of opportunity for policy change.

### 2006–2009

After the SARS outbreak, significant changes came to the public health system in Canada, bolstering pandemic preparedness and the ability to monitor emerging disease [26]. When an arbitration board faced a union challenging mandatory influenza vaccination, this time in British Columbia, the arbitrator sided with the employer noting a requirement for general immunization in the union collective agreement. In this case the employee had choices: vaccination, anti-viral medication, or to be off work without pay. The arbitrator ruled that the choices, which were not dealt with in the St. Peter`s decision, meant there was no violation of the Charter under section 7. An important note in the decision summary was the rationale that preventing the spread of influenza to vulnerable populations is clear and was agreed upon by both the employer and the union, which allowed for a realignment of the policies stream with problems and politics streams.

May 2009 saw the first wave of the H1N1 pandemic influenza strain. By July 2009, the Public Health Agency of Canada reported it still did not have a plan in place to manage HCWs who refused the seasonal influenza vaccine [27]. The seasonal vaccine did not include protection from the novel H1N1 strain and thus, worldwide production of a vaccine was implemented. Stories of production delays, low vaccine supplies, and rationing caused long lines at vaccination clinics and resulted in public anxiety [28]. Even after this pandemic experience, uptake of the vaccine by HCWs the following season remained low [23]. The H1N1 pandemic could have resulted in the opening of a new policy window, but with the relatively mild nature of the H1N1 strain, the mandatory vaccination issue did not gain adequate or sustained public attention (politics stream), closing another window of opportunity.

### 2010–2015

A long-awaited economic appraisal of Ontario’s UIIP was published in 2010 [21]. Based on a measure of quality-adjusted life years, the study concluded that UIIP was cost-effective and might be considered for adoption in other provinces and countries with similar populations, health care models, and influenza rates. This evidence bolstered Ontario’s influenza vaccination program and may have served as a catalyst to support further expansion of the program to include mandatory HCW vaccination, however, it was not sufficient to make mandatory vaccination a politically acceptable option.

In other jurisdictions, most notably the US, consideration of mandatory vaccination for HCWs was gaining momentum. A large US health care employer had published their successful program requiring influenza immunization for employment [14]. In Canada, the province of British Columbia initiated a task force in 2011 to investigate and recommend plans for mandatory immunization programs [29]. The task force visited nine US sites with programs in place before making recommendations in 2012 to the provincial government.

Pressure on the Ontario government to act intensified as both the evidence and prominent organizations supported mandatory HCW vaccination. For instance, the Influenza Surveillance Protocol for Ontario Hospitals was revised and included the National Advisory Committee on Immunization statement that “… refusal of health care workers who have direct patient contact to be immunized against influenza implies failure in their duty of care to their patients [30].” The Association of Medical Microbiology and Infectious Disease also presented a position paper supporting mandatory immunization acknowledging that all efforts at voluntary programs had failed [19]. Public Health Ontario, The Canadian Medical Association Journal, and The American Academy of Pediatrics joined in the call for mandatory immunization [31].

In July 2012, the British Columbia government announced a new policy that would require either influenza immunization or the wearing of a surgical mask for all HCWs during influenza season when in direct patient contact. By October 2012 the Health Sciences Association of British Columbia union had filed a grievance citing privacy issues relating to the Freedom of Information and Privacy Act (FIPA) and violations of the Human Rights Code, the Charter of Rights and Freedoms, and their collective union agreement [29]. Facing an election the following spring, the incumbent government did not repeal the policy but announced that it would wait for the outcome of the arbitration hearing and that workers who did not comply in the 2012–13 season would not face disciplinary action [32]. The government revised and softened the policy in July before going to arbitration [29]. The decision was released in October and the arbitrator had sided with the employer. It was determined the policy aligned with FIPA and there were no violations to the union agreement, the Charter of Rights and Freedoms or the Human Rights Code.

This court ruling provided the Ontario government, and health care organizations, with greater assurances that movement in the direction of mandatory vaccination for HCWs could be a viable policy option subject to HCWs being given some limited alternative to vaccination. Despite this, the Ontario government did not step forward with a consistent policy for the province. In an effort to fill this vacuum, numerous high profile Ontario hospitals announced plans to implement a mandatory “vaccinate or mask” policy that would require HCWs who chose not to get vaccinated to wear a mask during the influenza season [33]. Even though the Canadian Nurses’ Association supported mandatory influenza vaccinations for HCWs, the Ontario Nurses’ Association filed a grievance in opposition to the “vaccinate or mask” policy adopted by several Ontario hospitals. Following several weeks of testimony, the arbitrator found that the policy was unreasonable (policies stream) [34]. Concerns identified included that wearing a mask revealed personal health information about the HCWs decision not to vaccinate, masks were not effective, and the policy was designed to coerce HCWs [34]. This ruling leaves Ontario hospitals with no clear policy direction for the foreseeable future.

## Conclusions

By analysing the progress of mandatory HCW immunization through the lens of Kingdon’s framework, the separate streams of problems, politics, and policies can be seen to converge and diverge repeatedly over an extended period (policy windows have opened and closed several times). Since the implementation of UIIP in Ontario, there have been several potential opportunities to pursue a mandatory HCW vaccination policy. In each instance a technically feasible solution was available (policies stream). However, despite the policy's importance—protecting the public—the problem’s prominence (problems stream) and the political environment (politics stream) only aligned with the policies stream for a very short period of time. During these periods there was inadequate support for the issue to remain on the government’s decision agenda long enough to result in the adoption of a new policy. In part, this seems to have been due to other problems gaining prominence and displacing the issue of mandatory vaccination of HCWs from a priority position. In addition, each time this issue gained prominence, opposition lobby groups reacted, making the proposed solution less politically acceptable.

Although the call for mandatory HCW vaccination has withstood some legal challenges—including to the Charter of Rights and Freedoms, The Freedom of Information and Privacy Act, and the Human Rights Code—there remains small but committed groups who continue to oppose such a policy. As a result, it seems highly unlikely that a mandatory HCW vaccination policy will be adopted by the Ontario government until perception of the problem’s importance is sufficient to overcome the political opposition to implementing a solution.

## Abbreviations

FIPA: Freedom of information and privacy act
HCWs: health care workers
SARS: Severe acute respiratory syndrome
UIIP: Universal influenza immunization program
WHO: World Health Organization
